# Molecular simulations of SSTR2 dynamics and interaction with ligands

**DOI:** 10.1038/s41598-023-31823-1

**Published:** 2023-03-23

**Authors:** Silvia Gervasoni, Camilla Guccione, Viviana Fanti, Andrea Bosin, Giancarlo Cappellini, Bruno Golosio, Paolo Ruggerone, Giuliano Malloci

**Affiliations:** 1 Department of Physics, University of Cagliari, 09042 Monserrato (Cagliari), Italy; 2 Istituto Nazionale di Fisica Nucleare, Sezione di Cagliari, 09042 Monserrato (Cagliari), Italy

**Keywords:** Molecular modelling, Computational models, Cancer, Computational science, Computational chemistry, Structure-based drug design

## Abstract

The cyclic peptide hormone somatostatin regulates physiological processes involved in growth and metabolism, through its binding to G-protein coupled somatostatin receptors. The isoform 2 (SSTR2) is of particular relevance for the therapy of neuroendocrine tumours for which different analogues to somatostatin are currently in clinical use. We present an extensive and systematic computational study on the dynamics of SSTR2 in three different states: active agonist-bound, inactive antagonist-bound and *apo* inactive. We exploited the recent burst of SSTR2 experimental structures to perform μs-long multi-copy molecular dynamics simulations to sample conformational changes of the receptor and rationalize its binding to different ligands (the agonists somatostatin and octreotide, and the antagonist CYN154806). Our findings suggest that the *apo* form is more flexible compared to the *holo* ones, and confirm that the extracellular loop 2 closes upon the agonist octreotide but not upon the antagonist CYN154806. Based on interaction fingerprint analyses and free energy calculations, we found that all peptides similarly interact with residues buried into the binding pocket. Conversely, specific patterns of interactions are found with residues located in the external portion of the pocket, at the basis of the extracellular loops, particularly distinguishing the agonists from the antagonist. This study will help in the design of new somatostatin-based compounds for theranostics of neuroendocrine tumours.

## Introduction

**Figure 1:**
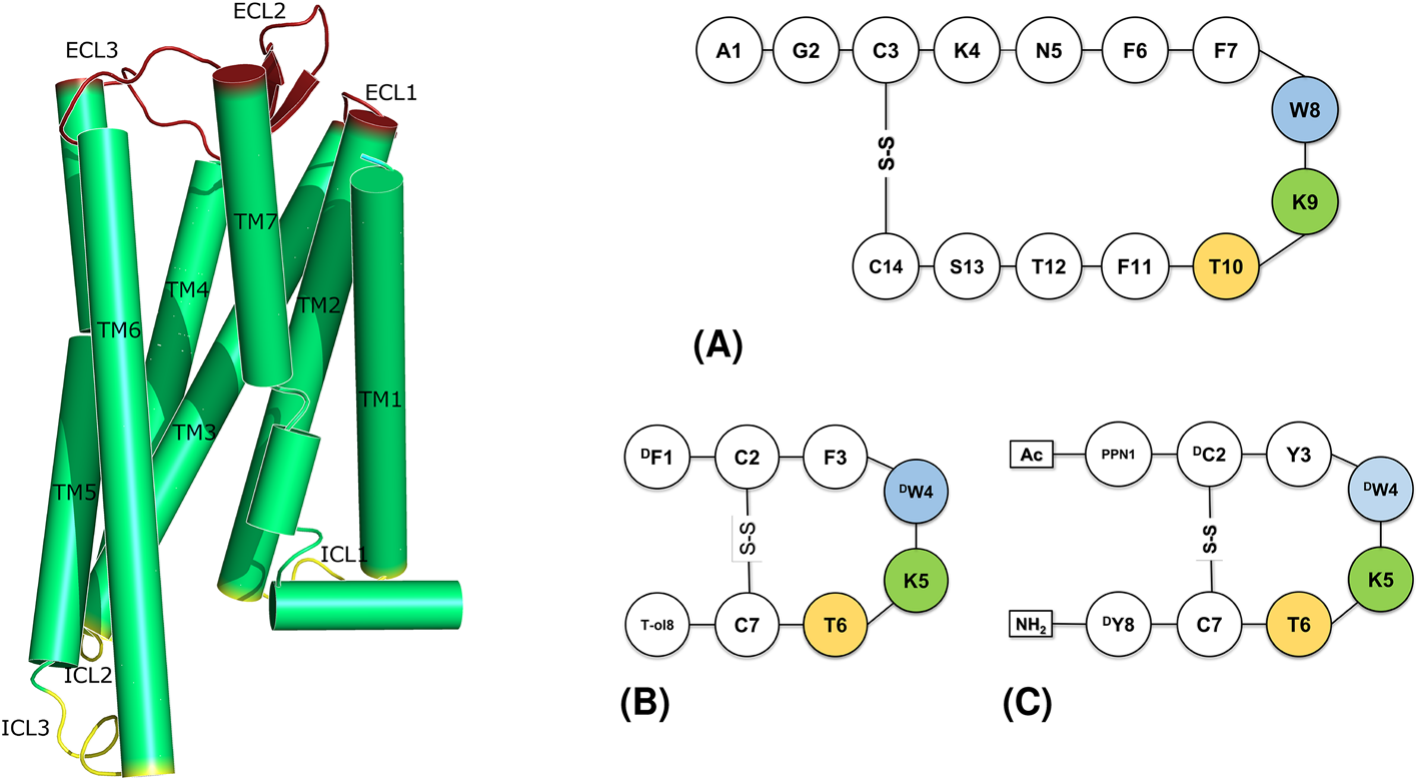
Left panel: Schematic representation of GPCR structure and main domains. Transmembrane helices (green): TM1 = A44$^{1.33}$-R70$^{1.59}$, TM2 = I77$^{2.38}$-A104$^{2.65}$, TM3 = K112$^{3.22}$-V145$^{3.55}$, TM4 = I150$^{4.33}$-A181$^{4.64}$, TM5 = G202$^{5.32}$-S237$^{5.67}$, TM6 = R245$^{6.24}$-V280$^{6.59}$, TM7 = P288$^{7.29}$-L315$^{7.56}$. Extracellular loops (red): ECL1 = L105-G111, ECL2 = G182-S201, ECL3 = S281-T287. Intracellular loops (yellow): ICL1 = Y71-T76, ICL2 = H146-P149, ICL3 = V238-S244. Right panel: 2D sketches of SSTR2 agonists: (A) SST14 and (B) octreotide (T-ol = threoninol), and (**C**) the antagonist CYN154806 (Ac = acetyl group, PPN = Phe-NO$_2$). Conserved residues are highlighted.

Somatostatin is a cyclic disulphide bond-containing hormone expressed in two splicing variants of 14 (SST14) and 28 amino acids. The former is the predominant form in the brain, while the latter is found primary in the gut^1,2^. Somatostatin plays a crucial role in regulating the release of different hormones such as insulin, growth hormone and secretin, through its inhibitory activity^3,4^. SST is an agonist of somatostatin receptors (SSTRs), which belong to class A G-protein coupled receptors (GPCRs). Most GPCRs present seven transmembrane alpha helices (TM1-7), three extracellular (ECL1-3) and three intracellular (ICL1-3) loops (Fig. 1, left panel. The Ballesteros-Weinstein numbering scheme for class A GPCRs is adopted throughout the paper^5^). SSTRs are coupled with inhibitory G-protein (i.e., G$_i$ or G$_0$)^6^, and can be divided into five families, from 1 to 5, among which the isoform 2 is the most expressed in human neuroendocrine tumours (NETs)^7,8^. Such an over-expression makes SSTR2 an important target for both anti-tumour therapy and diagnostic (i.e., theranostic)^9^. As a result, the development of drugs targeting SSTR2 is of great interest and a few molecules are in clinical use. Generally, these compounds mimic the structure of the endogenous agonist SST14^10^, and can be either peptides (e.g., octreotide^11^, lanreotide, pasireotide^12^, Fig. 1, right panel) or non-peptides (e.g., L-054,522 and L-054,264^13,14^). SST14 has a short half-life (< 3 min) and lacks selectivity towards the five SSTR isoforms. Differently, octreotide and lanreotide show higher affinity towards SSTR2 and a longer half-life (2 and 1 h, respectively)^10,15,16^. The non-peptide/peptidomimetic analogues of SST14 have shown increased half-life and potency towards specific SSTR isoforms as well^17^. Furthermore, they can function as agonist or antagonist^18,19^. Noteworthy, various studies have shown that antagonists have favourable pharmacokinetic profiles and better tumour visualization compared to agonists, thanks to their ability to bind multiple conformational states of SSTR2, despite poor internalization rates^19,20^.

Given the great interest in development of SST14 analogues, several studies addressing the structural basis of ligand binding to SSTR2 have been recently published. First, computational works that exploited homology modelling^21–23^. Then, some experimental structures started to appear. Robertson and co-workers released the first cryo-EM structures of SSTR2 bound with SST14 and octreotide^24^. New structures soon followed, also in complex with other ligands^14,15,25,26^ and one in the *apo* inactive form^27^ (Table S1). An important feature of the SSTR2 structure (common to other GPCRs) is the high flexibility of ECL2^28^, that was found completely open when in complex with SST14, folded down with octreotide, and in a middle position in the *apo* form^24^. Furthermore, all transmembrane helices engage rearrangements when moving from inactive to active states of the receptor. In particular, TM6 shows a hallmark outward movement^29^, TM5 is a common switch that moves closer to TM7, stabilizing TM6^30^, and TM3 is a hub for stabilization^31^.

The main pharmacophore features characterizing the SSTR2 peptide ligands are represented by (1) a $\beta$-sheet shape, at which tip are located (2) an aromatic group (often a tryptophan residue), and (3) a basic positively charged moiety (such as a lysine residue). This portion is inserted into the cavity of the transmembrane pocket, while the other part of the molecule faces the external loops of the receptor^14^. The increasing availability of experimental structures of SSTR2 has shed light on the structural features of the receptor and on the flexible elements that play a crucial role in the interaction with ligands. Molecular dynamics (MD) simulations performed by Robertson and co-workers^24^ for *apo*-SSTR2 revealed that ECL2 does not spontaneously fold over the orthosteric site, while it closes upon the binding pocket when SSTR2 was in complex with octreotide. However, an in-depth characterization of the dynamics of SSTR2 in the inactive antagonist-bound form in comparison with the *apo*-inactive and active agonist-bound ones is needed to achieve a more complete picture.

In this work, we performed MD simulations of SSTR2 in both *apo* and *holo* forms and explored the binding modes of the complexed ligands. We focused on the endogenous ligand SST14 and its peptide analogues octreotide (OCT) and CYN154806 (CYN), behaving as agonist and antagonist, respectively. We thoroughly quantified the opening and closing movements of ECL2. Similarly, following previous studies on GPCR dynamics, we determined structural metrics able to discriminate between active (i.e., structures solved in complex with the G-protein and an agonist ligand) and inactive (i.e., structures solved without the G-protein both in the *apo* and in the antagonist-bound forms) conformations of SSTR2. In the active and inactive conformations, the receptor is able to trigger or not the intracellular signal, respectively. Our results confirm that, as expected, the *apo* form of SSTR2 is characterised by a higher flexibility compared to the *holo* forms. We found that OCT induces the closure of ECL2, confirming previous findings, while the loop does not close upon CYN, despite their similar steric hindrance. Combination of binding free energies calculations and interaction fingerprints evaluations reveals peculiar behaviors of the three ligands in terms of intra- and inter-molecular interactions.

## Results and Discussion

To explore the dynamics of SSTR2 in different conformations and to elucidate agonist and antagonist binding at the molecular level, we performed multi-copy MD simulations, with an overall simulation time of 5 μs per system. In detail, we simulated the receptor in the active and inactive states. Conformational clusters extracted from MD simulations provided the basis for further characterizations both in energetic terms and patterns of detailed interactions.

### The binding of ligand stabilizes SSTR2 loops

Given the well-known challenge of GPCR experimental resolution^32–35^, the systems selected for this work were obtained using stabilizing agents: SSTR2-SST14 and SSTR2-OCT were in complex with the G-protein, SSTR2 *apo* with a nanobody, and SSTR2-CYN was expressed as a chimera. For this reason, we first assessed the overall stability of the systems during the MD simulations. For computational time reasons, we performed the MD simulations without including the G-protein. Since the allosteric effect of the G-protein is expected to have an impact on major conformational rearrangements, it is safe to assume that the lack of the G-protein in our plain μs-long MD simulations did not affect much the system structural features under investigation. Indeed, the average RMSD values of the backbone C$\alpha$ atoms (Fig. S1) were all below 2.5 Å, suggesting that the removal of the stabilizing agents did not alter the main conformation of the systems. Consistently, the RMSF of the backbone C$\alpha$ atoms (Fig. S2) reached the highest values for the intra and extracellular loops, while the transmembrane helices were highly stable in all cases. As expected, ECL2, which is the longest loop and interacts with the ligands, and ICL3, which is responsible for the recognition of the G-protein, were the most flexible domains, generally reaching or exceeding an RMSF value of 4 Å. However, we found some differences in loops movements through the four systems: the *apo* structure showed the largest loops flexibility (especially ECL2 and ECL3) compared to the other systems, while the SSTR2-SST14 complex is the one with the less flexible loops. These results agree with experimental findings for a generic GPCR, according to which its *apo* structure is highly unstable and prone to easily explore different conformational states, whereas the binding with a ligand stabilizes an active conformation of the receptor^32,36^. In particular, our data suggest that the endogenous ligand SST14 is more stabilizing than the synthetic agonist OCT and antagonist CYN (mean values of the backbone C$\alpha$ atoms RMSF integral for SST14 = 279 Å, OCT = 294 Å, CYN = 305 Å, *apo* = 307 Å).

### ECL2 closes upon octreotide but not CYN154806

**Figure 2:**
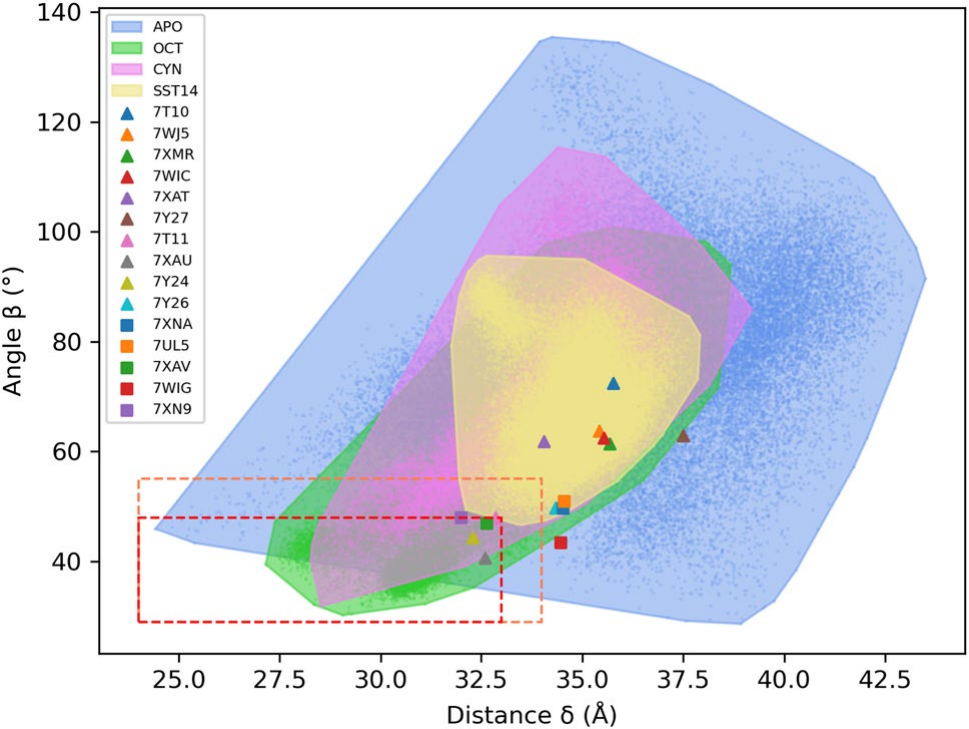
ECL2 opening and closing movements. Each point of the plot refers to a frame of the MD trajectories. The results of SST14, OCT, CYN and *apo* structures are coloured in yellow, green, magenta and blue, respectively. Threshold values of distance and angle are indicated. The red box includes the frames in which the ECL2 is in the closed conformation, the orange box indicates the border between open and closed conformations. The distance values range from 24 to 44 Å, the angle values from 29$^\circ$ to 135$^\circ$. The areas enclose all the frames (dots) corresponding to the four systems. The triangles and squares represent the experimental structures of SSTR2 (list of all experimental structures in Table S1).

The ECL2 is known to be highly flexible and its closure upon the binding pocket is believed to facilitate the interaction with ligands^37,38^. The RMSF values discussed above highlighted the involvement of this loop in the main differences between the dynamics of the four systems. Therefore, we analyzed in detail the opening and closing movements of ECL2 during the MD simulations. To this aim, we identified two geometric variables describing ECL2 movements. We computed (1) the distance $\delta$ between the loop tip (center of mass of Q187, W188, G189 C$\alpha$ atoms) and the center of mass of the seven TM helices C$\alpha$ atoms, and (2) the angle $\beta$ between the W188 C$\alpha$ atom, the base of ECL2 (center of mass of A181 and I195 C$\alpha$ atoms), and the base of ECL3 (center of mass of S281 and P288 C$\alpha$ atoms) (Fig. S3). These two values were computed for all MD frames of each replica (saved every 1 ps), and combined in the scatter plot shown in Fig. 2. After a careful visual inspection of the MD trajectories, we identified threshold values of distance $\delta$ and angle $\beta$ allowing for a distinction between open and close configuration of the ECL2 loop (i.e., $24< \delta < 33$ Å and 29 $< \beta < 48^\circ$ for the close conformation). According to these values, as shown in Fig. 2, the experimental structures in complex with OCT (7XAU and 7T11), lanreotide (7XAV), and L-054,522 (7XN9) have a closed loop, while all the others feature an open loop. These results agree with previous findings^24^.

Each point in Fig. 2 refers to one MD frame, which is associated with single values of distance and angle. All the frames included in the red/orange boxes are those in which ECL2 is in the closed conformation according to the above criteria. It is interesting to compare the corresponding percentages of occurrence in the four cases: 28.2% for SSTR2-OCT, 1.5% for SSTR2-CYN, 0.1% for the *apo* form and $\sim$0% for SST14.

The distribution of points in the plot mirrors the RMSF trend: SSTR2-SST14 and SSTR2-CYN show the less flexible loop, followed by SSTR2-OCT in which $\sim$28% of the frames present a closed ECL2. In the case of SST14, ECL2 cannot close upon the binding pocket due to the steric hindrance of the ligand (mean Connolly surface area^39^ collected during the MD trajectories for SST14, OCT and CYN: 1273 ± 26 Å$^2$, 887 ± 30 Å$^2$, and 895 ± 28 Å$^2$, respectively). Whereas, the marked difference between the loop motions in the complexes with OCT and CYN can be found in the different interaction patterns between the two peptides (see below). The points corresponding to the *apo* form are the most scattered, reinforcing the higher flexibility compared to the *holo* structures, and suggesting that ligands play a key role in the stabilization of ECL2.

### Key features distinguishing active from inactive conformations of SSTR2

GPCRs are able to trigger the intracellular signal through the presence of the G-protein (or arrestins), and thanks to both microswitches of aminoacids^29^ and major conformational changes. These receptors are indeed able to assume multi-conformational states, that can be stabilized by the presence of a ligand^28^.

**Figure 3:**
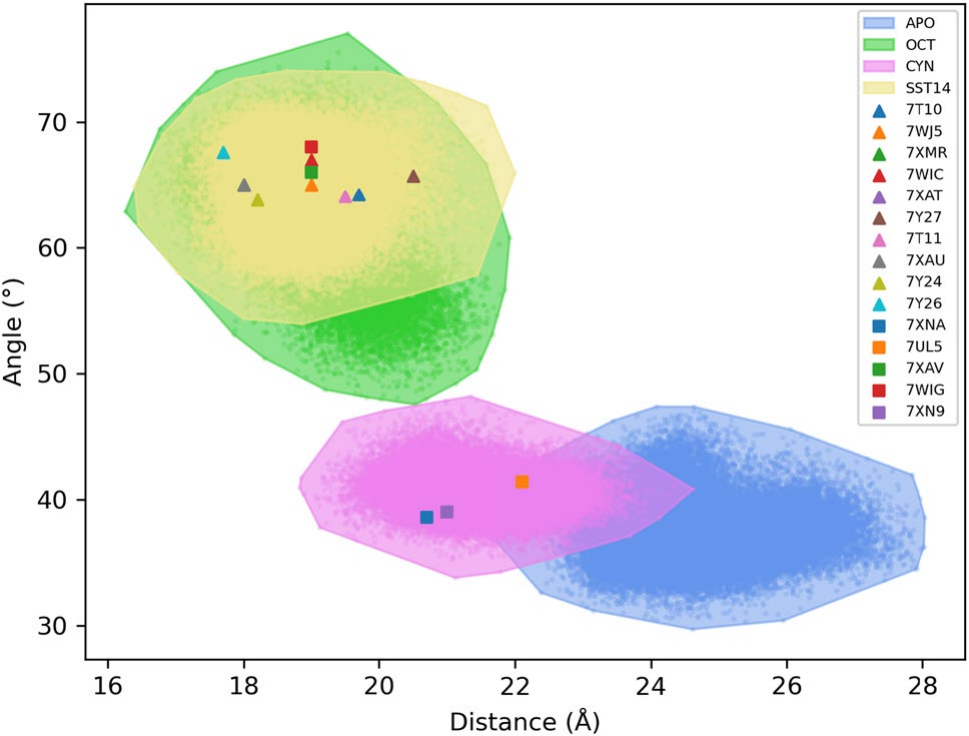
Scatter plot separating active from inactive conformations in SSTR2. The yellow, green, magenta and blue dots refer to each frame of the SSTR2-SST14, SSTR2-OCT, SSTR2-CYN and SSTR2 *apo* trajectories, respectively. The areas around dots represent the frame extension. The triangles and squares represent the experimental structures of SSTR2 (list of all experimental structures in Table S1).

The transition from inactive to active form of class A GPCRs involves the movement of TM5, TM6 and TM7: an outward displacement of TM5 and TM6, an inward movement of TM7 at the intracellular side, and an additional inwards shift of TM5 and TM7 at the extracellular side^29–31^. In a recent work, Lu and co-workers identified two geometric variables able to distinguish between active and inactive forms of the class A GPCR angiotensin II^40^, suggesting that these parameters could be likewise measured for other GPCRs of the same class. On this ground, we computed these parameters on our MD trajectories, by considering (1) the distance between the C$\alpha$ atoms of C225$^{5.55}$ and S305$^{7.46}$, and (2) the angle between the C$\alpha$ atoms of T255$^{6.34}$, C268$^{6.47}$ and I80$^{2.41}$ (Fig. S4). The first quantity accounts for the conformational changes of TM5 and TM7 (active $< \sim$19 Å), while the second one reflects the outward movement of TM6 (active > $\sim$45$^\circ$). Figure 3 reports a scatter plot in which each point represents a frame of the MD trajectories associated with the distance and angle values. The active conformations of SSTR2 in complex with SST14 and OCT, and the inactive *apo* conformation are well clustered into two distinct regions of the plot, spanning from 16 to 22 Å and from 48$^\circ$ to 77$^\circ$ (active), and from 22 to 28 Å and from 30$^\circ$ to 47$^\circ$ (inactive). On the contrary, the inactive conformation of the receptor in complex with the antagonist CYN is located in an intermediate region, ranging from 19 to 25 Å and from 34$^\circ$ to 48$^\circ$, partly overlapping with the *apo* region and, at the same time, extending towards the active one.

Interestingly, the experimental structures corresponding to the active complexes are located in the center of the active region, while the experimental conformation of the *apo* form (i.e., PDB 7UL5) is located at the edge of the inactive area. These results further confirm the high instability of SSTR2 in the *apo* state, that during the 5 μs of MD simulations explored multiple conformations, moving away from that reported in the cryoEM structure. The only exception is represented by the PDB structure 7XN9 (i.e., SSTR2 in complex with a non-peptide agonist L-054,522), which is located in the antagonist/inactive region. It is noteworthy that, this is the only SSTR2-agonist complex in which the receptor is not coupled with the G-protein, which can explain its peculiar behaviour.

### Intramolecular interactions of peptides influence the binding with SSTR2

The ligands share the same cyclic $\beta$-sheet structure, presenting the characteristic residues Trp ($^D$Trp for OCT and CYN) and Lys that interact at the bottom of the binding pocket with I177$^{4.60}$, F208$^{5.38}$ and D122$^{3.32}$, Q126$^{3.36}$, Y302$^{7.43}$, respectively. The terminal portions of all peptides are in contact with the extracellular loops of the receptor, a structural feature that is believed to confer the isoform selectivity^14^, together with some elements of the TM bundle^41^. Despite the generally high flexibility of peptides, all ligands firmly interact with SSTR2, as reported by the average RMSD values below 3 Å (Fig. S5). Although these values indicate a great stability of the binding modes, the RMSF analysis revealed that some portions of the ligands are still highly flexible (Fig. S6). In SST14, besides a high RMSF at the N-terminal, F7 fluctuated significantly during the simulations (Fig. S6A). Similarly, in OCT the N-terminal threoninol and the F3 reached the highest RMSF values (Fig. S6B). CYN shows a high stability, with the exception of one replica, in which it reaches an average RMSD of 4.1 ± 0.4 Å (Fig. S5C). This high value is due to the loss of the $\pi$-$\pi$ intramolecular interaction between Y3 and $^D$Y8, which are the residues that fluctuated the most (Figs. S6C, S7). As expected, in all ligands the conserved ($^D$)Trp and Lys residues were highly stable in all trajectories.

The ligands were also stabilized by intramolecular interactions between the side chains atoms. In particular, F11 of SST14 interacts with F6 and N5, and in turn N5 interacts with T12, while $^D$F1 of OCT interacts with F3 and C7 (Fig. S8A,B). The intramolecular interactions of CYN involved Y3 and $^D$Y8, which is favoured by the presence of a *cis* amide bond that forces the conformation of Y3 towards $^D$Y8, engaging a persistent aromatic interaction. Further CYN intramolecular interactions can be found between $^D$W4 with the N-terminal (i.e., Ac-PPN1-$^D$C2), T6 with $^D$C2 and Y3, and PPN1 with C7 (Fig. S8C).

**Figure 4:**
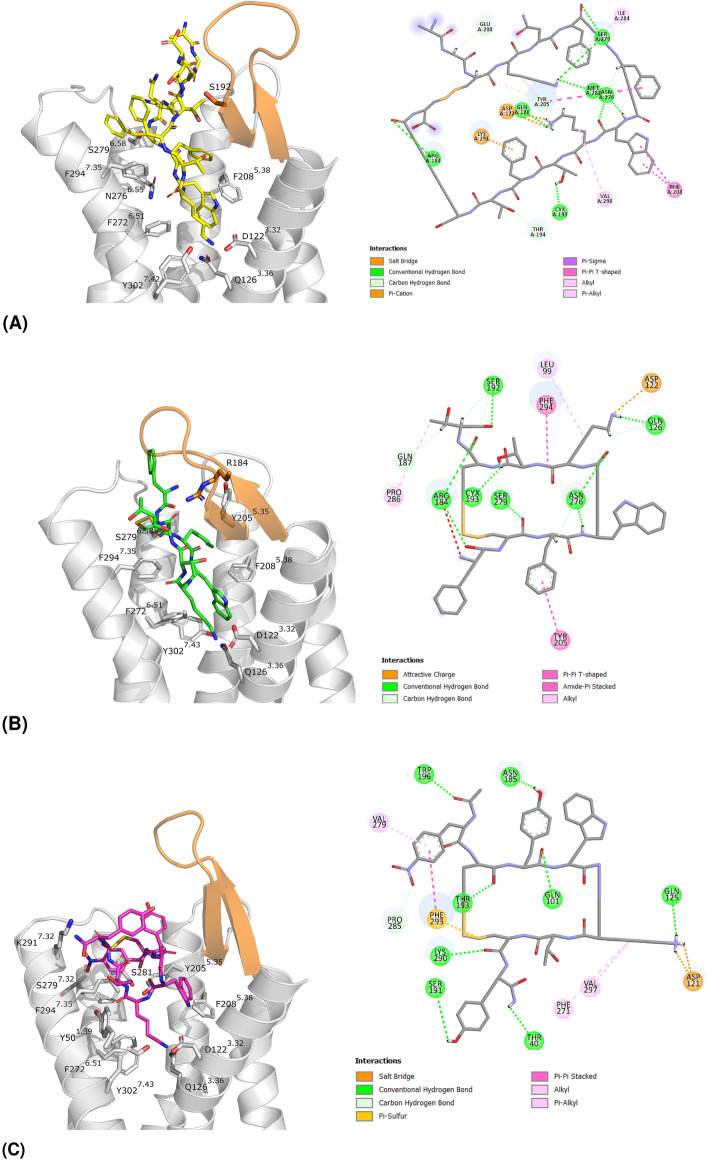
Representatives of the most populated cluster of (**A**) SSTR2-SST14 (64.5%) in yellow, (**B**) SSTR2-OCT (42.9%) in green, (**C**) SSTR2-CYN (79.1%) in magenta. On the left: 3D representation, ECL2 is coloured in orange, the interacting residues are represented in sticks. On the right: 2D interaction diagram, generated by Discovery Studio^42^.

The five trajectories of each SSTR2 *holo* system were first concatenated and then subjected to a cluster analysis (see “Methods”, Table S2). Figure 4 shows the representatives of the most populated cluster for each system, to highlight the different binding modes. In agreement with the low RMSD/F values, these conformations are comparable to those of the corresponding experimental structures (i.e., RMSD of heavy atoms with respect to the experimental structure of SSTR2-SST14: 2.5 Å, SSTR2-OCT: 1.7 Å, SSTR2-CYN: 2.3 Å, Table S2).

Interestingly, $\sim$79% of the frames of SSTR2-CYN trajectories belong to the most populated cluster, which is the most similar to the experimental binding mode. While the second cluster represents the conformation lacking the $\pi$-$\pi$ interaction between Y3 and $^D$Y8 (Fig. S7, RMSD 4.6 Å).

A binding free energy analysis on the conformational clusters extracted from the trajectories was performed using the MM-GBSA method (see “Methods”). Table S2 lists the average values, weighted on the cluster population. The endogenous ligand SST14 is the compound that binds SSTR2 with the higher affinity (− 90.1 ± 10.7 kcal/mol), while OCT and CYN show a comparable binding affinity (− 70.0 ± 12.4 kcal/mol and − 73.4 ± 10.2 kcal/mol, respectively). These results are consistent with experimental data reporting an IC$_{50}$ value of 0.2 nM for SST14, and 0.6 nM for OCT^43^.

### SSTR2 agonists and antagonist show different interaction patterns

**Figure 5:**
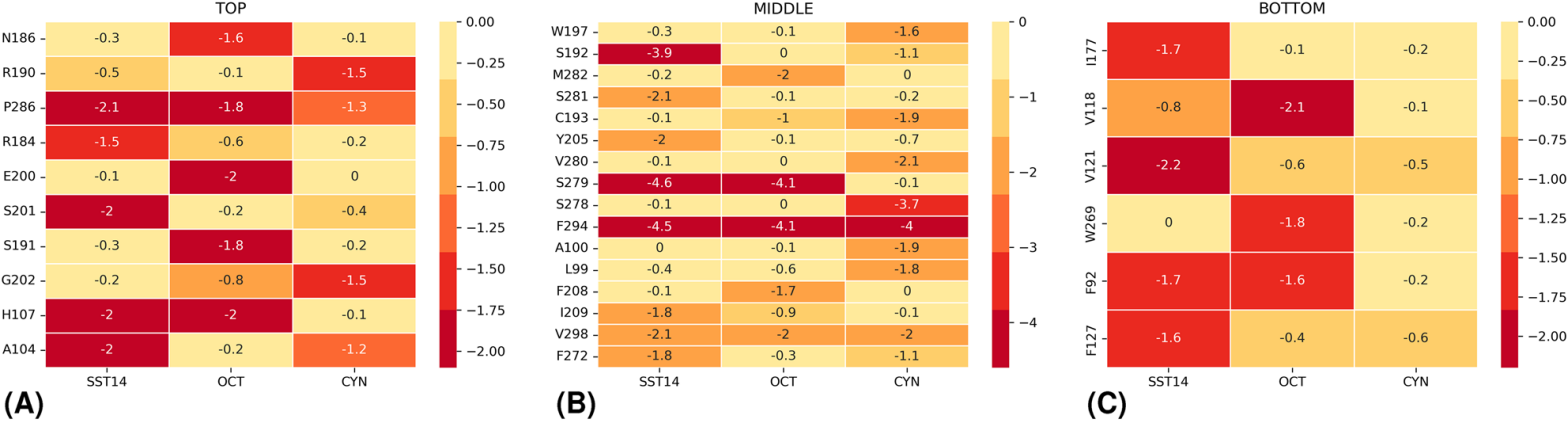
Partial contribution of SSTR2 residues to the MM-GBSA binding free energy (kcal/mol) for SST14, OCT and CYN. Values are reported in kcal/mol according to the pocket regions: (**A**) top, (**B**) middle, (**C**) bottom and ordered according to their spatial localization. Only residues in which at least one contribution reached an energy value of − 1.0 kcal/mol are reported. The cells are colored from the highest to the lowest values of the binding free energy (i.e., from yellow to red).

We divided the binding pocket into three different regions, according to the spatial distribution of protein residues: bottom, middle and top. The complete list of residues and their spatial distribution is reported in Table S3. Then, we computed for the three peptides the partial contribution to the binding free energy of residues located in each region (Fig. 5).

All peptides strongly interact with F294$^{7.35}$, V298$^{7.39}$ (middle, TM7) and P286 (top, ECL3). SST14 and OCT share a similar interaction pattern, both interacting with F92$^{2.53}$, S279$^{6.58}$ and H107. However, some differences through ligands can be found. SST14 interacts with V121$^{3.31}$, Y205$^{5.35}$, S281$^{6.60}$ and S201 with an energy of 1.3 kcal/mol (or more) lower than OCT and CYN. Similarly, interaction with V118$^{3.28}$, M282 and E200 is stronger for OCT, and with L99$^{2.60}$, A100$^{2.61}$ and V280$^{6.59}$ for CYN.

**Figure 6:**
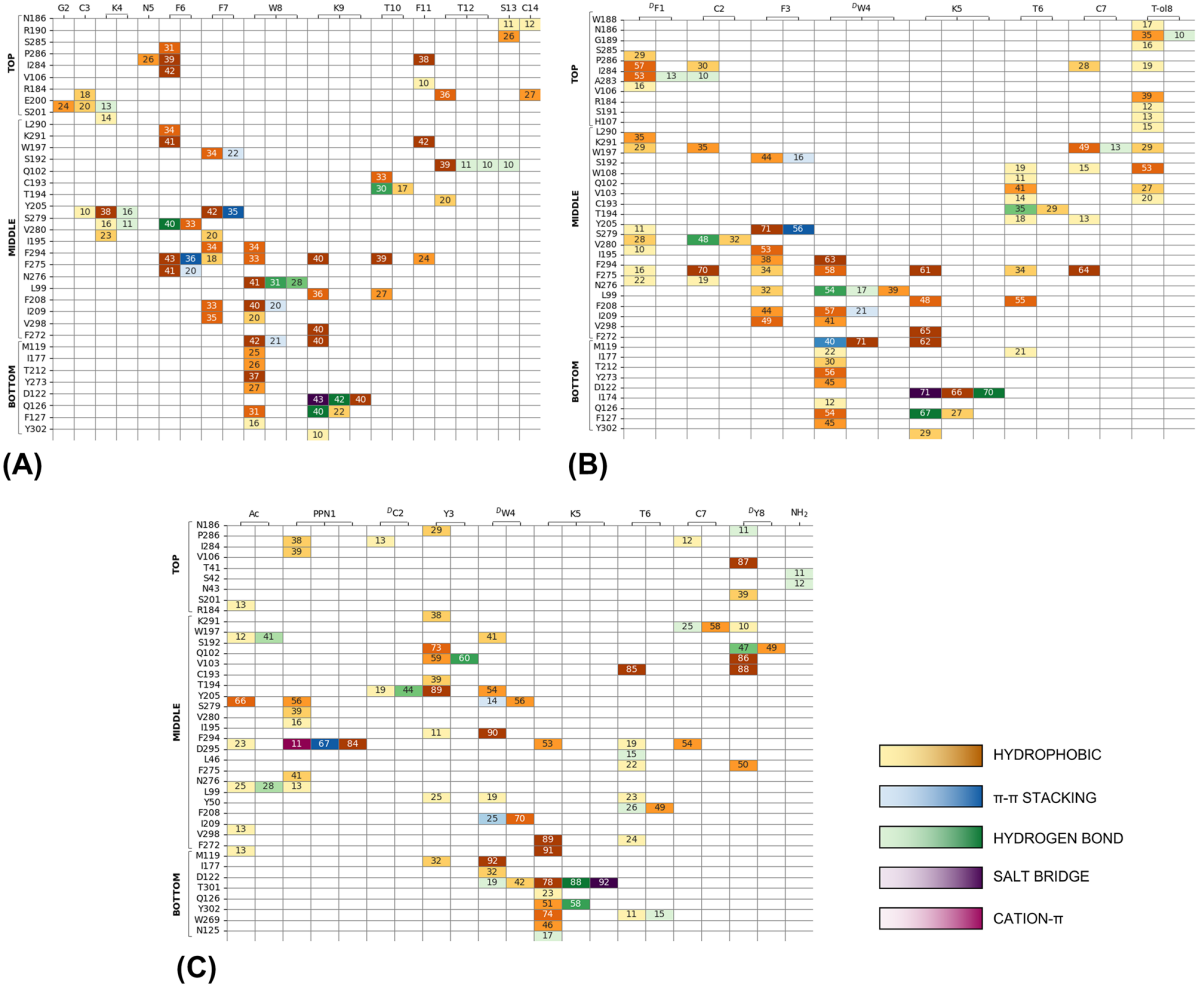
Interaction fingerprints for (**A**) SSTR2-SST14, (**B**) SSTR2-OCT, (**C**) SSTR2-CYN. The numbers indicate the persistence of interaction (%) between the residue of SSTR2 (vertical axis) and that of the ligand (horizontal axis). Darker colors correspond to higher persistences. Interactions are coloured according to their type: yellow to orange for hydrophobic, blue for $\pi$-$\pi$ stacking, green for hydrogen bonds, violet for salt bridges, and magenta for cation-$\pi$. Only interactions equal or greater than 10% are reported. The residues of SSTR2 are divided according to the pocket regions (i.e., top, middle, bottom) and ordered according to their spatial localization.

To further investigate the key SSTR2/peptides contacts we performed an interaction fingerprint analysis (Fig. 6). Generally, most interactions are hydrophobic, involving also aromatic side chains. SST14 interacts mainly (> 30%) with residues belonging to ECL3 (I284, S285, P286) in the top region, and with residues belonging to TM5 (Y205$^{5.35}$, F208$^{5.38}$), TM6 (F272$^{6.51}$, F275$^{6.54}$, N276$^{6.55}$, S279$^{6.58}$), and TM7 (K291$^{7.32}$, F294$^{7.35}$, V298$^{7.39}$) in the middle region. Similarly, OCT interacts mainly (>50%) with domains in the top and middle regions: ECL3 (I284, P286), TM5 (Y205$^{5.35}$, F208$^{5.38}$), TM7 (F294$^{7.35}$, V298$^{7.39}$), TM6 (F272$^{6.51}$, N276$^{6.55}$, V280$^{6.59}$) common to SST14, and I195 in the ECL2. Conversely, fewer overlaps are observed for CYN: TM5 (Y205$^{5.35}$, F208$^{5.38}$), TM6 (F272$^{6.51}$) and TM7 (K291$^{7.32}$, F294$^{7.35}$). In this latter case the major interactions (> 50%) involve residues peculiar to this peptide: ECL1 (V106), ECL2 (S192, T194, I195), TM2 (Q102$^{2.63}$, V103$^{2.64}$).

Furthermore, the Lys residue in each ligand (K9 for SST14 and K5 for both OCT and CYN) stably interacts with D122$^{3.32}$, Q126$^{3.36}$ and Y302$^{7.43}$, as already reported in literature based on structural data^25^. Differently, we found that the Trp residue (W8 for SST14 and ^D^W4 for both OCT and CYN) maintains the interaction with I177$^{4.60}$, looses that with F208$^{5.38}$, and gains an additional one, Q126$^{3.36}$ (for SST14 and OCT) or D122$^{3.32}$ (for CYN).

Interestingly, this last difference between SST14/OCT and CYN is not the only one: almost all residues interacting with SST14 are also common to OCT (33 out of 36, $\sim$92%). Whereas, CYN shared with SST14 and OCT the $\sim$81% and $\sim$71% of the interacting residues, respectively. Furthermore, by looking at Fig. 5A,B, a similar interaction pattern characterizing SST14 and OCT can be clearly identified throughout the top, middle and bottom regions. Differently, only the bottom region looks comparable in CYN (Fig. 5C).

Noteworthy, the patterns shown in Fig. 5 can contribute explaining the different dynamical behaviour of ECL2 in SSTR2/OCT and SSTR2/CYN systems. By comparing the interaction fingerprints we found the main differences in residues belonging to ECL2, TM1 and TM2, which interact the most with CYN, and in ECL3 and TM6, which on the contrary mostly interact with OCT. This means that OCT is mainly located in the pocket region opposite to that of the bottom of ECL2, resulting in much more freedom of movement for the loop. Differently, CYN resides most of the simulation time near the bottom of ECL2, thus hindering the closure of the loop (Fig. S9).

## Conclusions

Somatostatin receptors, especially the isoform SSTR2, represent a prominent target for NET theranostics. Lately, the burst of experimental structures of SSTR2 has given the chance to study the conformational features of this receptor and, as a consequence, further explore the key determinants of interaction with the endogenous ligand SST14 and its synthetic analogues. In this work we performed multi-copy μs-long MD simulations of SSTR2 in three different forms: active agonist-bound, inactive antagonist-bound, and *apo* inactive. Our results show that the *apo* state is characterized by a higher flexibility compared to the *holo* states, in particular for the EC and IC loops. Thanks to this higher flexibility, through our MD simulations we were able to extensively sample conformations of the *apo* form that differ to a certain extent from the experimental structure (see Figs. 2, 3). Furthermore, by monitoring the opening and closing movements of ECL2, we found that this loop is able to close upon OCT, but it mainly remains open in the *apo* form, with the endogenous ligand SST14 and, surprisingly, with the antagonist ligand CYN. In particular, ECL2 closes very rarely upon CYN, although its number of residues and surface area are like those of OCT. This suggests that steric hindrance alone is not the only feature driving ECL2 closure, but the specific residues involved in the ligand/SSTR2 interaction are crucial for orienting the ligands in a position that allows more space for loop movements. The binding free energy analysis combined with the interaction fingerprints reveal that all peptides similarly interact with the buried residues of the binding pocket (e.g., D122$^{3.32}$, Q126$^{3.36}$, F294$^{7.35}$). However, differences were found in the interaction patterns with residues located in the external portion of the pocket (i.e., at the ECLs). These findings confirm that the well-defined pharmacophore region of the pocket is essential for ligand binding, while the interactions with the external residues appear to discriminate between the agonists and the antagonist. Further molecular-level studies for a larger set of compounds are needed to assess the above findings. Furthermore, the two agonists SST14 and OCT shared a similar intra-molecular pattern of interaction, which is different for the antagonist CYN thus reflecting a markedly distinct binding to the receptor.

To the best of our knowledge, this is the first computational study systematically exploring the dynamics of SSTR2 in different conformational states, exploiting the recently released experimental structures. Our findings contribute to drug design efforts towards the discovery of new somatostatin-based compounds for theranostic of neuroendocrine tumours.

## Methods

The starting 3D structures of SSTR2 in complex with SST14, OCT, CYN and in the *apo* form were retrieved from the PDB IDs 7T10, 7T11^24^, 7XNA^14^ and 7UL5^27^. To include missing atoms each structure underwent structure refinement using Modeller10.2^44^. 7XNA was resolved as a chimeric structure comprising the stabilizing endo-1,4-beta-xylanase from *Niallia circulans* at the ICL3. We removed this portion and modelled the missing six residues (i.e., from S238 to G243). To reduce computational costs we did not include the G-protein in the structures. The ionization state of the residue side chains, the tautomeric states of histidine residues and the Asn/Gln flipping were checked by the H++ server^45^. The CHARMM-GUI server^46^ was used to embed the protein into a double layer of phosphatidyl choline (POPC, 70%) and cholesterol (30%)^47^. The system was inserted in an OPC water box^48^ and neutralized by adding K$^+$ and Cl$^-$ ions, reaching a 0.15 M concentration.

The AmberTools20 software^49^ was used to assign the force field ff19SB to the protein and to SST14^50^, lipid17 to POPC and cholesterol^51^, and the hydrogen mass repartition scheme^52^. OCT and CYN contain non-standard residues (i.e., $^D$F1, $^D$W4, threoninol for OCT and Phe-NO$_2$, $^D$C2, $^D$W4, $^D$Y8 for CYN), therefore we generated the parameters adopting the following procedure: (1) Gaussian16 (Revision A.03)^53^ was employed to compute the electrostatic potential of non-standard residues (B3LYP/6-31G** level of theory), (2) we fitted the atomic partial charges using Antechamber^54^ and the RESP method^55^, (3) the final topological files were created using the ff19SB force field and employing the prepgen and parmchk2 programs^49^. Since the nitro group contained in Phe-NO$_2$ of CYN is not parametrized in ff19SB, we added the corresponding parameters manually, according to the reference values reported in GAFF2^56^. The PDB structure of CYN reports a *cis* amide bond between Y3 and $^D$W4. To simulate this conformation, during the MD simulations we applied dihedral restraints to this bond by imposing the *cis* form (equilibrium dihedral= 0.0 ± 10.0$^\circ$, force constant= 50.0 kcal/mol/Å$^2$). The parm7 and rst7 files of the OCT and CYN are available in the Supporting materials.

Each system underwent an energy minimization combining the steepest-descent and the conjugated gradient algorithms and applying positional restraints on the protein and membrane atoms. Two steps of NVT and four of NPT equilibration followed the minimization, in which the positional restraints were incrementally reduced. We used the Langevin thermostat (1 ps$^{-1}$ as collision frequency) and the Berendsen barostat (1 Atm), a cutoff of 9 Å, the time step was incremented from 1 to 2 fs with the SHAKE algorithm^57^, the Particle Mesh Ewald method for long-range electrostatics^58^. The production run was carried on for 1 μs, using the NPT ensemble and 4 fs as a time step. Five replicas were generated for each system, resulting in a 5 μs overall simulation time. The MD simulations were conducted using the PMEMD module of Amber20^49^.

CPPTRAJ^59^ was used to perform the cluster analysis of MD trajectories. In detail, a hierarchical algorithm^60^ was used to group the frames into four conformational clusters, according to the ligand RMSD.

Binding free energy calculations using the MM-GBSA method implemented in Amber20^61^ were applied to each cluster (see Supporting Information for additional details on the method). Interaction fingerprints were computed using the ProLIF Python library^62^ on all the frames of the MD trajectories. The numbers of interactions were combined for all replicas and converted into persistence of interactions (%).

## Supplementary Information

Supplementary Information.

## Data Availability

All data generated or analysed during this study are included in this published article and its supplementary information files. The raw files of the MD trajectories and of the corresponding input topology and coordinates files are freely available at Zenodo 10.5281/zenodo.7727861.

## References

[1] Günther T (2018). International Union of Basic and Clinical Pharmacology. CV. Somatostatin receptors: Structure, function, ligands, and new nomenclature. Pharmacol. Rev..

[2] Fani M, Mansi R, Nicolas GP, Wild D (2022). Radiolabeled somatostatin analogs—a continuously evolving class of radiopharmaceuticals. Cancers.

[3] Börzsei R (2022). Exploration of somatostatin binding mechanism to somatostatin receptor subtype 4. Int. J. Mol. Sci..

[4] Shamsi BH, Chatoo M, Xu XK, Xu X, Chen XQ (2021). Versatile functions of somatostatin and somatostatin receptors in the gastrointestinal system. Front. Endocrinol..

[5] Isberg V (2015). Generic GPCR residue numbers—aligning topology maps minding the gaps. Trends Pharmacol. Sci..

[6] Rocheville M (2000). Subtypes of the somatostatin receptor assemble as functional homo- and heterodimers. J. Biol. Chem..

[7] Elf A-K (2021). Evaluation of SSTR2 expression in SI-NETs and relation to overall survival after PRRT. Cancers.

[8] Qian ZR (2016). Association between somatostatin receptor expression and clinical outcomes in neuroendocrine tumors. Pancreas.

[9] Heidari P (2018). Somatostatin receptor type 2 as a radiotheranostic PET reporter gene for oncologic interventions. Theranostics.

[10] Gomes-Porras M, Cárdenas-Salas J, Álvarez Escolá C (2020). Somatostatin analogs in clinical practice: A review. Int. J. Mol. Sci..

[11] Paragliola RM, Salvatori R (2018). Novel somatostatin receptor ligands therapies for acromegaly. Front. Endocrinol..

[12] Vitale G (2018). Pasireotide in the treatment of neuroendocrine tumors: A review of the literature. Endocr. Relat. Cancer.

[13] Chen L-N (2022). Structures of the endogenous peptide- and selective non-peptide agonist-bound SSTR2 signaling complexes. Cell Res..

[14] Zhao W (2022). Structural insights into ligand recognition and selectivity of somatostatin receptors. Cell Res..

[15] Bo Q (2022). Structural insights into the activation of somatostatin receptor 2 by cyclic SST analogues. Cell Discov..

[16] Harris AG (1994). Somatostatin and somatostatin analogues: Pharmacokinetics and pharmacodynamic effects. Gut.

[17] Yang L (1998). Synthesis and biological activities of potent peptidomimetics selective for somatostatin receptor subtype 2. Proc. Natl. Acad. Sci. USA.

[18] Karimian N (2013). Somatostatin receptor type 2 antagonism improves glucagon counterregulation in biobreeding diabetic rats. Diabetes.

[19] Fani M, Nicolas GP, Wild D (2017). Somatostatin receptor antagonists for imaging and therapy. J. Nucl. Med..

[20] Koustoulidou S (2022). Synthesis and evaluation of two long-acting SSTR2 antagonists for radionuclide therapy of neuroendocrine tumors. Pharmaceuticals.

[21] Cai Z (2014). 64Cu-labeled somatostatin analogues conjugated with cross-bridged phosphonate-based chelators via strain-promoted click chemistry for PET imaging: In silico through in vivo studies. J. Med. Chem..

[22] Nagarajan SK, Babu S, Sohn H, Devaraju P, Madhavan T (2019). Toward a better understanding of the interaction between somatostatin receptor 2 and its ligands: A structural characterization study using molecular dynamics and conceptual density functional theory. J. Biomol. Struct. Dyn..

[23] Nagarajan SK (2021). Understanding the influence of lipid bilayers and ligand molecules in determining the conformational dynamics of somatostatin receptor 2. Sci. Rep..

[24] Robertson MJ, Meyerowitz JG, Panova O, Borrelli K, Skiniotis G (2022). Plasticity in ligand recognition at somatostatin receptors. Nat. Struct. Mol. Biol..

[25] Heo Y (2022). Cryo-EM structure of the human somatostatin receptor 2 complex with its agonist somatostatin delineates the ligand-binding specificity. Elife.

[26] Chen S, Teng X, Zheng S (2022). Molecular basis for the selective G protein signaling of somatostatin receptors. Nat. Chem. Biol..

[27] Robertson MJ (2022). Structure determination of inactive-state GPCRs with a universal nanobody. Nat. Struct. Mol. Biol..

[28] Latorraca NR, Venkatakrishnan AJ, Dror RO (2017). GPCR dynamics: Structures in motion. Chem. Rev..

[29] Zhou Q (2019). Common activation mechanism of class A GPCRs. Elife.

[30] Hauser AS (2021). GPCR activation mechanisms across classes and macro/microscales. Nat. Struct. Mol. Biol..

[31] Weis WI, Kobilka BK (2018). The molecular basis of G protein-coupled receptor activation. Annu. Rev. Biochem..

[32] Heydenreich FM, Vuckovic Z, Matkovic M, Veprintsèv DB (2015). Stabilization of G protein-coupled receptors by point mutations. Front. Pharmacol..

[33] Grisshammer R (2020). The quest for high-resolution G protein-coupled receptor-G protein structures. Proc. Natl. Acad. Sci. USA.

[34] Grisshammer R (2017). New approaches towards the understanding of integral membrane proteins: A structural perspective on G protein-coupled receptors. Protein Sci..

[35] Salom D, Padayatti PS, Palczewski K (2013). Crystallization of G protein-coupled receptors. Methods Cell Biol..

[36] Wacker D, Stevens RC, Roth BL (2017). How ligands illuminate GPCR molecular pharmacology. Cell.

[37] Nicoli A, Dunkel A, Giorgino T, de Graaf C, Pizio AD (2022). Classification model for the second extracellular loop of class A GPCRs. J. Chem. Inf. Model.

[38] Woolley MJ, Conner AC (2017). Understanding the common themes and diverse roles of the second extracellular loop (ECL2) of the GPCR super-family. Mol. Cell Endocrinol..

[39] Connolly ML (1983). Analytical molecular surface calculation. J. Appl. Cryst..

[40] Lu S (2021). Activation pathway of a G protein-coupled receptor uncovers conformational intermediates as targets for allosteric drug design. Nat. Commun..

[41] Lee SM (2015). Structural insights into ligand recognition and selectivity for classes A, B, and C GPCRs. Eur. J. Pharmacol..

[42] Systémes, B. D. Discovery Studio, 12.1.0. (Dassault Systémes, 2021).

[43] Anthony L, Freda PU (2009). From somatostatin to octreotide LAR: Evolution of a somatostatin analogue. Curr. Med. Res. Opin..

[44] Eswar N (2006). Comparative protein structure modeling using Modeller. Curr. Protoc. Bioinformatics.

[45] Gordon JC (2005). H++: A server for estimating pKas and adding missing hydrogens to macromolecules. Nucleic Acids Res..

[46] Jo S, Kim T, Iyer VG, Im W (2008). CHARMM-GUI: A web-based graphical user interface for CHARMM. J. Comput. Chem..

[47] Saeedimasine M, Montanino A, Kleiven S, Villa A (2019). Role of lipid composition on the structural and mechanical features of axonal membranes: A molecular simulation study. Sci. Rep..

[48] Izadi S, Anandakrishnan R, Onufriev AV (2014). Building water models: A different approach. J. Phys. Chem. Lett..

[49] Case, D. *et al.* Amber (University of California, 2022).

[50] Tian C (2020). ff19SB: Amino-acid-specific protein backbone parameters trained against quantum mechanics energy surfaces in solution. J. Chem. Theory Comput..

[51] Gould, I., Skjevik, A., Dickson, C., Madej, B. & Walker, R. Lipid17: A comprehensive AMBER force field for the simulation of zwitterionic and anionic lipids. *in prep.* (2018).

[52] Hopkins CW, Grand SL, Walker RC, Roitberg AE (2015). Long-time-step molecular dynamics through hydrogen mass repartitioning. J. Chem. Theory Comput..

[53] Frisch, M. J. *et al.* Gaussian 16, (Gaussian, Inc.).

[54] Wang J, Wang W, Kollman PA, Case DA (2006). Automatic atom type and bond type perception in molecular mechanical calculations. J. Mol. Graph. Model..

[55] Singh UC, Kollman PA (1984). An approach to computing electrostatic charges for molecules. J. Comput. Chem..

[56] Wang J, Wolf RM, Caldwell JW, Kollman PA, Case DA (2004). Development and testing of a general amber force field. J. Comput. Chem..

[57] Kräutler V, van Gunsteren WF, Hünenberger PH (2001). A fast SHAKE algorithm to solve distance constraint equations for small molecules in molecular dynamics simulations. J. Comput. Chem..

[58] Darden T, York D, Pedersen L (1993). Particle mesh Ewald: An N$$\cdot$$log(N) method for Ewald sums in large systems. J. Chem. Phys..

[59] Roe DR, Cheatham TE (2013). PTRAJ and CPPTRAJ: Software for processing and analysis of molecular dynamics trajectory data. J. Chem. Theory Comput..

[60] Shao J, Tanner SW, Thompson N, Cheatham TE (2007). Clustering molecular dynamics trajectories: 1. Characterizing the performance of different clustering algorithms. J. Chem. Theory Comput..

[61] Miller BR (2012). MMPBSA.py: An efficient program for end-state free energy calculations. J. Chem. Theory Comput..

[62] Bouysset C, Fiorucci S (2021). ProLIF: A library to encode molecular interactions as fingerprints. J. Cheminform..

