# Supplementary material for: Molecular simulations of SSTR2 dynamics and interaction with ligands
Source: Sci Rep. 2023 Mar 23;13:4768. doi: 10.1038/s41598-023-31823-1 (PMC10036620; doi:10.1038/s41598-023-31823-1)
Supplement: Supplementary file 1 — Supplementary Information. [file 41598_2023_31823_MOESM1_ESM.pdf]

Molecular simulations of SSTR2 dynamics and interaction with ligands

Silvia Gervasoni[1], Camilla Guccione[1], Viviana Fanti[1,2], Andrea Bosin[1], Giancarlo Cappellini[1], Bruno Golosio[1,2], Paolo Ruggerone[1], and Giuliano Malloci[1,*]

[1]University of Cagliari, Department of Physics, Monserrato (Cagliari), I-09042, Italy
[2]Istituto Nazionale di Fisica Nucleare, Sezione di Cagliari, Monserrato (Cagliari), I-09042, Italy
[*]giuliano.malloci@dsf.unica.it

SUPPORTING INFORMATION

PDB ID	Technique	Resolution (Å)	Ligand	G-protein coupled	Full human
7XMR[1]	Cryo-EM	3.10	SST14	Yes	Yes
7WIC[2]	Cryo-EM	2.80	SST14	Yes	Yes
7XAT[3]	Cryo-EM	2.85	SST14	Yes	Chimeric
7WJ5[4]	Cryo-EM	3.72	SST14	Yes	Yes
7T10[5]	Cryo-EM	2.50	SST14	Yes	Yes
7Y27[6]	Cryo-EM	3.48	SST14	Yes	Yes
7XAV[3]	Cryo-EM	2.87	LAN	Yes	Chimeric
7XAU[3]	Cryo-EM	2.97	OCT	Yes	Chimeric
7T11[5]	Cryo-EM	2.70	OCT	Yes	Yes
7Y24[6]	Cryo-EM	3.25	OCT	Yes	Yes
7Y26[6]	Cryo-EM	3.30	OCT	Yes	Yes
7XN9[1]	X-ray	2.60	L-054,522	No	Chimeric
7WIG[2]	Cryo-EM	2.70	L-054,264	Yes	Yes
7XNA[1]	X-ray	2.65	CYN 154806	No	Chimeric
7UL5[7]	Cryo-EM	3.10	*apo*	No	Yes

Table S1. SSTR2 available experimental structures (updated to 25/11/2022). Lanreotide agonist is indicated as LAN.

	SST14			OCT			CYN		
	%	RMSD	ΔG $\pm$ STD	%	RMSD	ΔG (kcal/mol) $\pm$ STD	%	RMSD	ΔG (kcal/mol) $\pm$ STD
c0	64.5	2.5	-92.2 $\pm$ 10.7	42.9	1.7	-70.9 $\pm$ 15.6	79.1	2.3	-74.7 $\pm$ 11.4
c1	26.4	2.9	-87.3 $\pm$ 10.2	36.5	1.8	-73.1 $\pm$ 10.2	10.4	4.6	-70.1 $\pm$ 5.8
c2	7.8	3.5	-87.3 $\pm$ 11.9	13.9	1.9	-63.3 $\pm$ 9.7	9.4	3.6	-67.6 $\pm$ 5.7
c3	1.3	2.5	-89.3 $\pm$ 12.2	6.7	1.4	-61.2 $\pm$ 9.1	1.1	2.9	-63.1 $\pm$ 6.5
Mean	2.7		-90.1 $\pm$ 10.7	1.7		-70.0 $\pm$ 12.4	2.7		-73.4 $\pm$ 10.2

Table S2. Cluster population (%), heavy atoms RMSD (Å) of the cluster representatives with respect to the starting experimental structure, and MM-GBSA binding free energies (kcal/mol) with the corresponding standard deviation. The average RMSD and binding free energies values are weighted on cluster population (Mean).

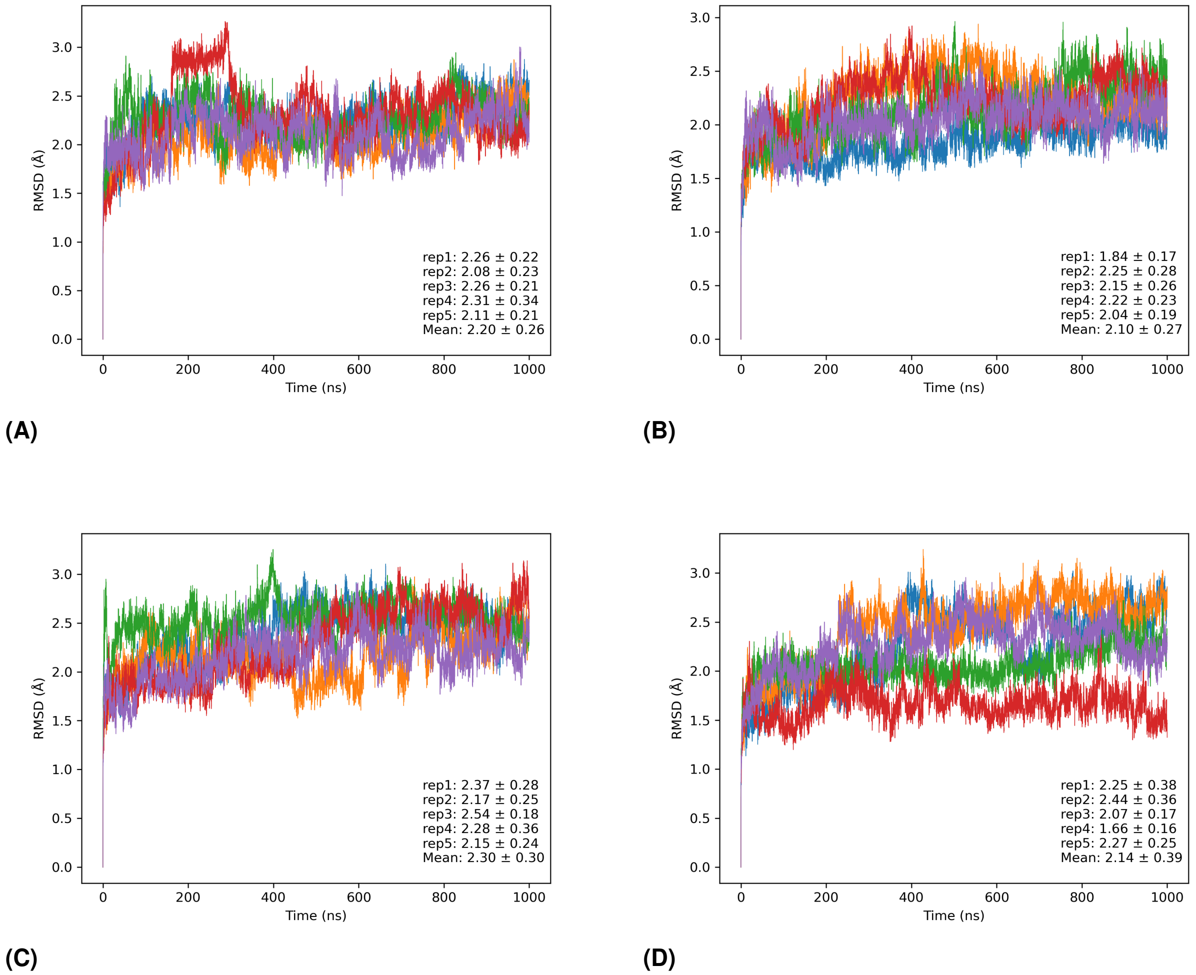

Figure S1. RMSD values of SSTR2 Cα atoms (Å), with respect to the first frame of the MD trajectory. RMSD values are reported for the four systems and the five replicas (blue, orange, green, red, and violet for replicas one to five). The average values are also reported, with the corresponding standard deviations. (A) SSTR2-SST14, (B) SSTR2-OCT, (C) SSTR2-CYN, (D) SSTR2-APO.

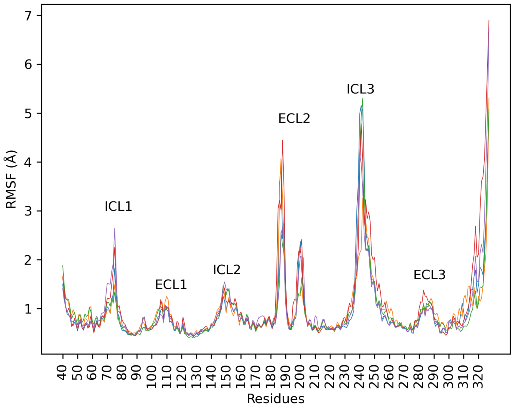

(A)

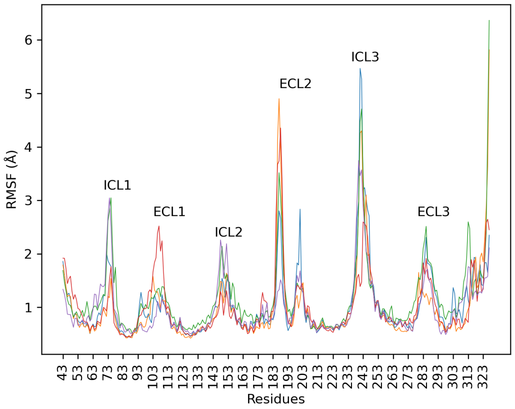

(B)

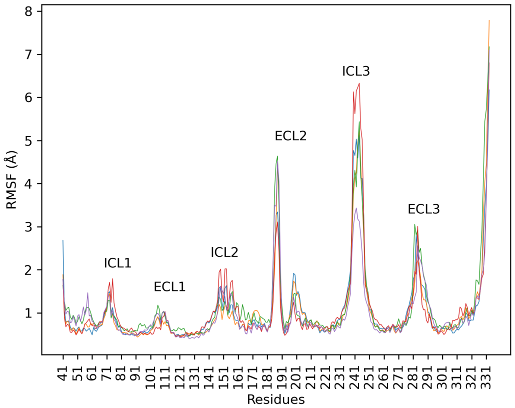

(C)

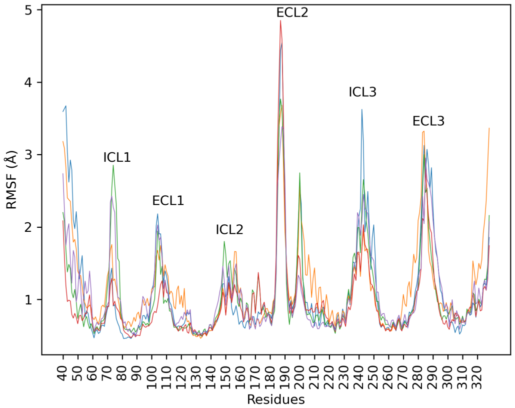

(D)

Figure S2. RMSF values of SSTR2 Cα atoms (Å). RMSF values are reported for the four systems and the five replicas (blue, orange, green, red, and violet for replicas one to five). (A) SSTR2-SST14, (B) SSTR2-OCT, (C) SSTR2-CYN, (D) SSTR2-APO.

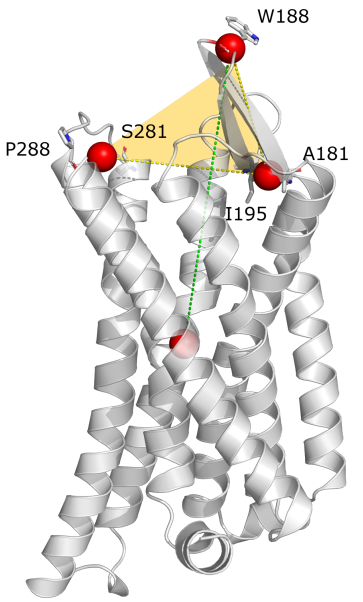

Figure S3. ECL2 opening and closing assessment. The green dotted line refers to the distance between the loop tip (center of mass of Q187, W188, G189 Cα atoms) and center of mass of the seven TM helices Cα atoms. The yellow dotted lines define the angular parameter β. The first segment connects the W188 Cα and the center of ECL2 baseline (center of mass of A181 and I195 Cα atoms), The other segment connects the latter point with the center of the ECL3 baseline (center of mass of S281 and P288 Cα atoms). The PDB ID 7T10 is shown as example.

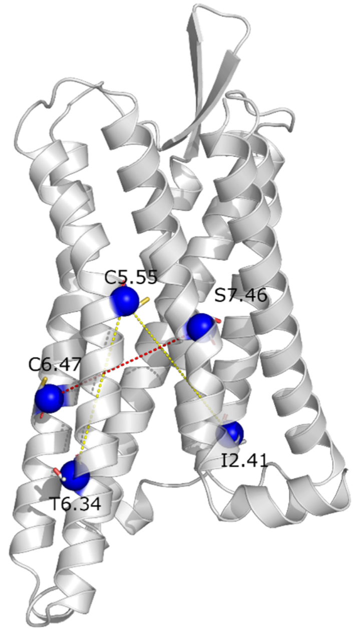

Figure S4. Key features distinguishing active from inactive conformations in SSTR2. The red dotted line represents the distance between the Cα atom of C225$^{5.55}$ and S305$^{7.46}$. The yellow dotted line represents the angle between the Cα atom of T255$^{6.34}$, C268$^{6.47}$ and I80$^{2.41}$. The PDB ID 7T10 is shown as example.

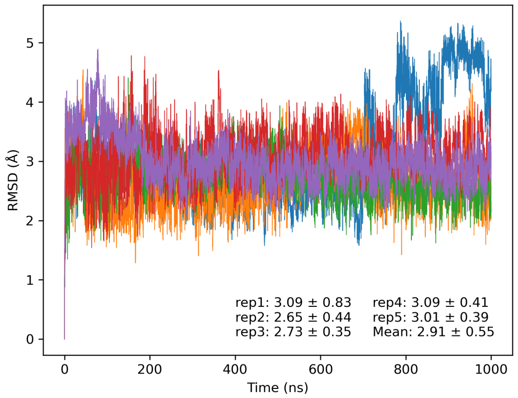

(A)

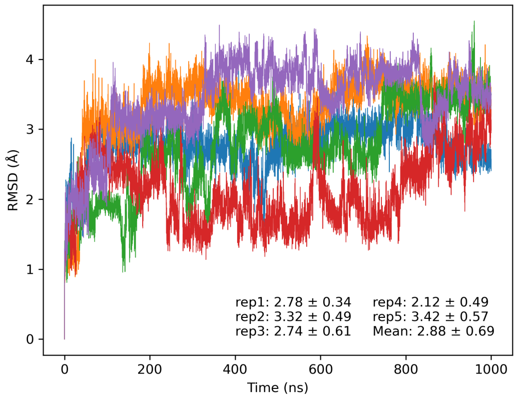

(B)

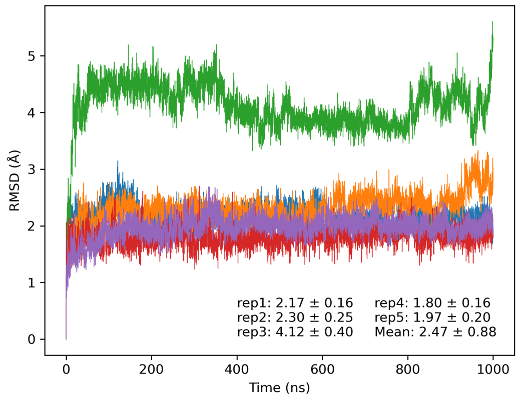

(C)

Figure S5. RMSD values (Å) of (A) SST14, (B) OCT, (C) CYN heavy atoms, with respect to the first frame of the MD trajectory. RMSD values are reported for the five replicas (blue, orange, green, red, and violet for replicas one to five) with the average values and the corresponding standard deviations.

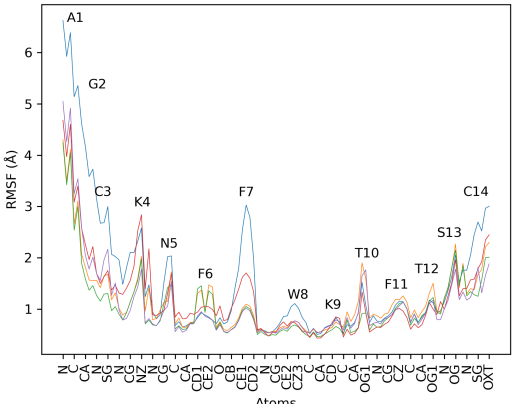

(A)

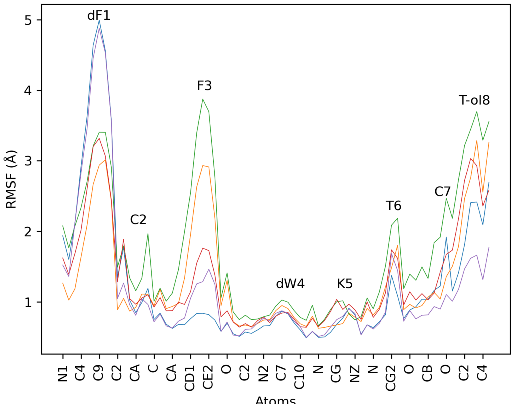

(B)

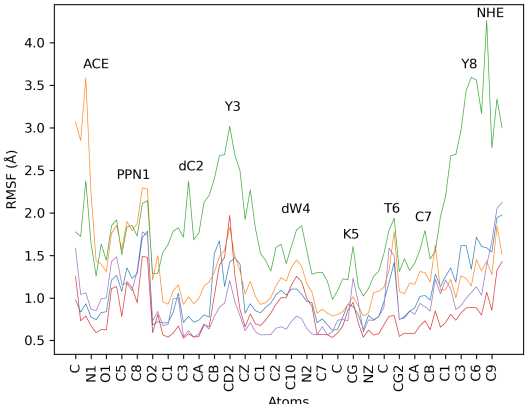

(C)

Figure S6. RMSF values (Å) of (A) SST14, (B) OCT, (C) CYN heavy atoms. RMSF values are reported for the five replicas (blue, orange, green, red, and violet for replicas one to five).

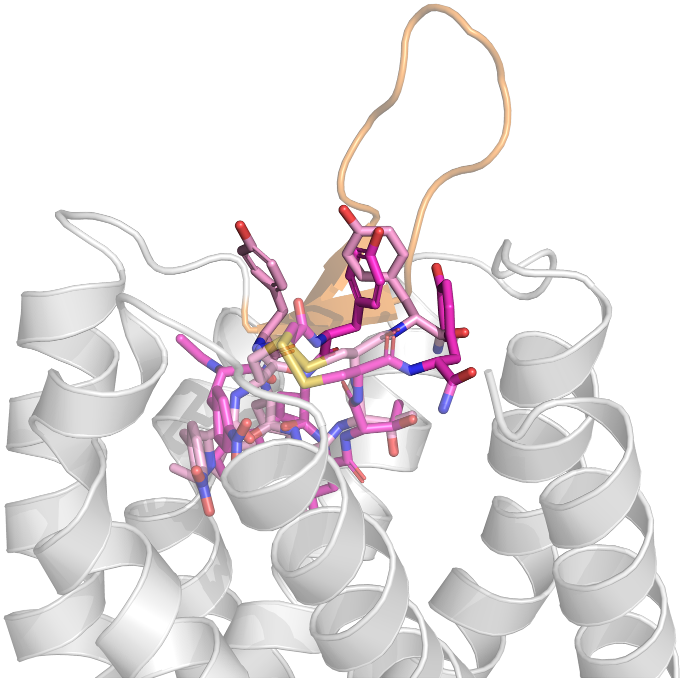

Figure S7. Superimposition of CYN representatives from cluster 0 (79.1%, magenta) and cluster 1 (10.4%, pink). In cluster 1 the π-π interaction between Y3 and ^{D}Y8 is lost.

	A1	G2	C3	K4	N5	F6	F7	T10	F11	T12	S13	C14
**A1**	--	0	0	0	0	0	0	0	0	0	0	0
**G2**	--	--	0	H	0	0	0	0	0	0	0	0
**C3**	--	--	--	0	H	0	0	0	0	H	0	0
**K4**	--	--	--	--	0	H AD	H	0	0	H	0	0
**N5**	--	--	--	--	--	0	H AD	0	H	AD H AD	0	H AD AD
**F6**	--	--	--	--	--	--	0	0	H P	0	0	0
**F7**	--	--	--	--	--	--	--	AD AD	0	0	0	0
**T10**	--	--	--	--	--	--	--	--	0	H AD	0	0
**F11**	--	--	--	--	--	--	--	--	--	0	H	0
**T12**	--	--	--	--	--	--	--	--	--	--	0	H AD
**S13**	--	--	--	--	--	--	--	--	--	--	--	0
**C14**	--	--	--	--	--	--	--	--	--	--	--	--

(A)

	^{D}F1	C2	F3	W4	T6	C7	T-ol8
**^{D}F1**	--	AD	H	0	0	H	H
**C2**	--	--	0	AD	H	0	0 0
**F3**	--	--	--	0	0	0	0 0
**W4**	--	--	--	--	0	0	0 0
**T6**	--	--	--	--	--	0	H AD
**C7**	--	--	--	--	--	--	0 0
**T-ol8**	--	--	--	--	--	--	--

(B)

	Ac	PPN1	^{D}C2	Y3	^{D}W4	K5	T6	C7	^{D}W8	NH$_2$
**Ac**	--	0	0	0	H	H	0 0	0	0 0	0
**PPN1**	--	--	0	0	H	H	0 0	H	0 0	0
**^{D}C2**	--	--	--	0	H	H	H	0	0 0	0
**Y3**	--	--	--	--	0	0	H AD	H	P H	0
**^{D}W4**	--	--	--	--	--	0	AD H	0	0 0	0
**K5**	--	--	--	--	--	--	0 0	H	0 0	0
**T6**	--	--	--	--	--	--	-- --	0	AD H	AD
**C7**	--	--	--	--	--	--	-- --	--	0 0	0
**^{D}Y8**	--	--	--	--	--	--	-- --	--	-- --	0
**NH$_2$**	--	--	--	--	--	--	-- --	--	-- --	--

(C)

Figure S8. Intra-interaction fingerprints for (A) SST14, (B) OCT, (C) CYN. Interactions are coloured according to their persistence (only values greater than 10%) from yellow to red. Interaction types are reported (H: hydrophobic, AD: H-bond acceptor/donor, P: π-π stacking).

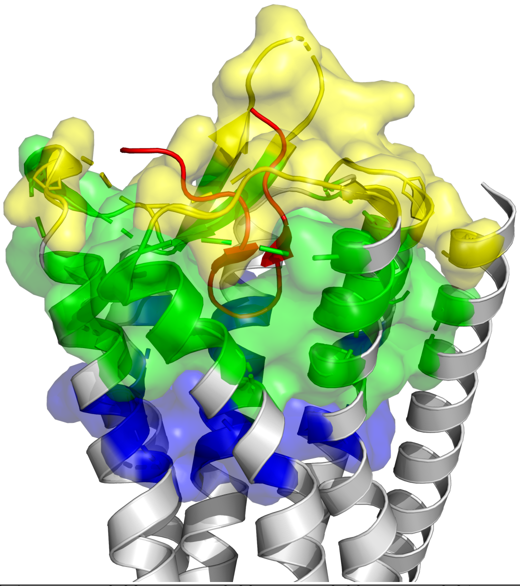

Bottom	F92$^{2.53}$, M93$^{2.54}$, L96$^{2.57}$, V118$^{3.28}$, M119$^{3.29}$, T120$^{3.30}$, V121$^{3.31}$, D122$^{3.32}$, G123$^{3.33}$, I124$^{3.34}$, N125$^{3.35}$, Q126$^{3.36}$, F127$^{3.37}$, I174$^{4.57}$, I177$^{4.60}$, Y211$^{5.41}$, T212$^{5.42}$, F213$^{5.43}$, G216$^{5.46}$, W269$^{6.39}$, Y273$^{6.43}$, T301$^{7.42}$, and Y302$^{7.43}$
Middle	L46$^{1.36}$, Y50$^{1.40}$, L99$^{2.60}$, A100$^{2.61}$, Q102$^{2.63}$, V103$^{2.64}$, W108^{ECL1}, F110^{ECL1}, A181$^{4.64}$, L183^{ECL2}, S192^{ECL2}, C193^{ECL2}, T194^{ECL2}, I195^{ECL2}, W197^{ECL2}, P198^{ECL2}, G199^{ECL2}, W204$^{5.34}$, Y205$^{5.35}$, T206$^{5.36}$, F208$^{5.38}$, I209$^{5.39}$, F272$^{6.51}$, F275$^{6.54}$, N276$^{6.55}$, V277$^{6.56}$, S278$^{6.57}$, S279$^{6.58}$, V280$^{6.59}$, S281^{ECL3}, M282^{ECL3}, L290$^{7.31}$, K291$^{7.32}$, G292$^{7.33}$, M293$^{7.34}$, F294$^{7.35}$, D295$^{7.36}$, V297$^{7.38}$, V298$^{7.39}$, and V299$^{7.40}$
Top	S42^{N-term}, N43^{N-term}, A104$^{2.65}$, L105^{ECL1}, V106^{ECL1}, H107^{ECL1}, R184^{ECL2}, S185^{ECL2}, N186^{ECL2}, Q187^{ECL2}, W188^{ECL2}, G189^{ECL2}, R190^{ECL2}, S191^{ECL2}, N196^{ECL2}, E200^{ECL2}, S201^{ECL2}, G202$^{5.32}$, A283$^{5.33}$, I284$^{5.34}$, S285$^{5.35}$, P286$^{5.36}$, T287$^{5.37}$, and P288$^{5.38}$

Table S3. Top panel: graphical representation of the three pocket regions: bottom in blue, middle in green, and top in yellow. The PDB ID 7T10 is shown as exemplification. SST14 is reported in red cartoon. Bottom panel: list of residues belonging to the three pocket regions.

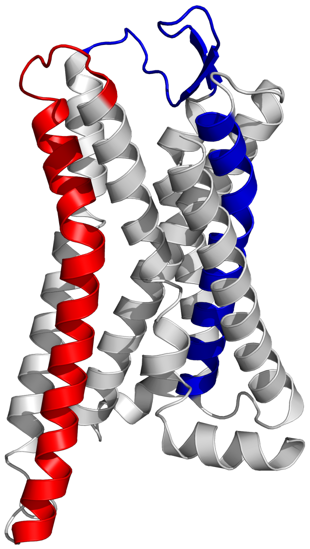

Figure S9. Most contacted SSTR2 segments by OCT (red: ECL3 and top of TM6) and CYN (blue: ECL2 and top of TM2). The structure of PDB ID 7T11 is used as exemplification.

MM-GBSA binding free energy calculation

The binding free energy of the peptides to SSTR2 was evaluated by means of the Molecular Mechanics – Generalized Born Surface Area (MM-GBSA) post-processing method[8] using the MMPBSA.py tool of the AmberTools package[9]. According to the MM-GBSA theory, the free energy of binding is evaluated through the following formula:

$$\Delta G = G_{com} - (G_{rec} + G_{lig}) \tag{1}$$

where G_{com}, G_{rec}, G_{lig} and are the absolute free energies of complex, receptor, and ligand, respectively, averaged over the equilibrium trajectory of the complex (single trajectory approach). According to these schemes, the free energy difference can be decomposed as:

$$\Delta G_b = \Delta G' - T\Delta S_{conf} \tag{2}$$

$$\Delta G_b = \Delta E_{MM} + \Delta G_{solv} - T\Delta S_{conf} \tag{3}$$

where ΔE_{MM} is the difference in the molecular mechanics energy, ΔG_{solv} is the solvation free energy, and $T\Delta S_{conf}$ is the solute conformational entropy. The first two terms were calculated with the following equations:

$$\Delta E_{MM} = \Delta E_{bond} + \Delta E_{angle} + \Delta E_{torsion} + \Delta E_{vdW} + \Delta E_{ele} \tag{4}$$

$$\Delta G_{solv} = \Delta G_{solv,p} + \Delta G_{solv,np} \tag{5}$$

ΔE_{MM} includes the molecular mechanics energy contributed by the bonded (ΔE_{bond}, ΔE_{angle}, and $\Delta E_{torsion}$) and non-bonded (ΔE_{vdW}, and ΔE_{ele}, calculated with no cutoff) terms of the force field. ΔG_{solv} is the solvation free energy, which can be modeled as the sum of an electrostatic contribution ($\Delta G_{solv,p}$, evaluated using the MM-GBSA approach) and a non-polar one ($\Delta G_{solv,np} = \gamma\Delta_{SA} + \beta$, proportional to the difference in solvent-exposed surface area, Δ_{SA}). In the MM-GBSA approach, the electrostatic solvation free energy was calculated using the implicit solvent model in ref.[10] (igb = 8 option in Amber20) in combination with mbondi3[11,12] and intrinsic radii. Partial charges were taken from the Amber20 ff19SB force field, and relative dielectric constants of 1 for solute and 78.4 for the solvent (0.15 M KCl water solution) were used. The non-polar contribution is approximated by the LCPO6 method implemented within the sander module of Amber20. In addition to being faster, the MM-GBSA approach provides an intrinsically easy way of decomposing the binding free energy into contributions from single atoms and residues. Solvation free energies were calculated on every cluster from each trajectory. The contribution of the configurational entropy of the solute has not been included.

References

1. Zhao, W. *et al.* Structural insights into ligand recognition and selectivity of somatostatin receptors. *Cell Res* **32**, 761–772, DOI: https://doi.org/10.1038/s41422-022-00679-x (2022).
2. Chen, L.-N. *et al.* Structures of the endogenous peptide- and selective non-peptide agonist-bound SSTR2 signaling complexes. *Cell Res* **32**, 785–788, DOI: https://doi.org/10.1038/s41422-022-00669-z (2022).
3. Bo, Q. *et al.* Structural insights into the activation of somatostatin receptor 2 by cyclic SST analogues. *Cell Discov* **8**, 47, DOI: https://doi.org/10.1038/s41421-022-00405-2 (2022).
4. Heo, Y. *et al.* Cryo-EM structure of the human somatostatin receptor 2 complex with its agonist somatostatin delineates the ligand-binding specificity. *Elife* **11**, e76823, DOI: https://doi.org/10.7554/eLife.76823 (2022).
5. Robertson, M. J., Meyerowitz, J. G., Panova, O., Borrelli, K. & Skiniotis, G. Plasticity in ligand recognition at somatostatin receptors. *Nat Struct Mol Biol* **29**, 210–217, DOI: https://doi.org/10.1038/s41594-022-00727-5 (2022).
6. Chen, S., Teng, X. & Zheng, S. Molecular basis for the selective G protein signaling of somatostatin receptors. *Nat Chem Biol* DOI: https://doi.org/10.1038/s41589-022-01130-3 (2022).
7. Robertson, M. J. *et al.* Structure determination of inactive-state GPCRs with a universal nanobody. *Nat Struct Mol Biol* DOI: https://doi.org/10.1038/s41594-022-00859-8 (2022).

8. Kollman, P. A. *et al.* Calculating Structures and Free Energies of Complex Molecules: Combining Molecular Mechanics and Continuum Models. *Acc Chem Res* **33**, 889–897, DOI: https://doi.org/10.1021/ar000033j (2000).

9. Case, D. *et al.* Amber 2022, University of California, San Francisco.

10. Shang, Y., Nguyen, H., Wickstrom, L., Okur, A. & Simmerling, C. Improving the Description of Salt Bridge Strength and Geometry in a Generalized Born Model. *J Mol Graph Model.* **29**, 676–684, DOI: https://doi.org/10.1016/j.jmgm.2010.11.013 (2011).

11. Bondi, A. Van der Waals Volumes and Radii. *J Phys Chem* **68**, 441–451, DOI: https://doi.org/10.1021/j100785a001 (1964).

12. Onufriev, A., Bashford, D. & Case, D. A. Exploring Protein Native States and LargeScale Conformational Changes with a Modified Generalized Born Model. *Proteins* **55**, 383–394, DOI: https://doi.org/10.1002/prot.20033 (2004).
